# Minimal immune response after transplantation of a cryopreserved human amniotic membrane on the ocular surface

**DOI:** 10.1016/j.mtbio.2026.102821

**Published:** 2026-01-17

**Authors:** Jean-Baptiste Baudey, Lauriana Solecki, Christophe Picard, Bastien Mathéaud, Pauline Jamain, Isabelle Jollet, Lucas Hubert, Alain Coaquette, Adeline Desanlis, Xavier Lafarge, Pascal Pedini, Florelle Gindraux

**Affiliations:** a Établissement Français du Sang Provence Alpes Côte d’Azur-Corse, Laboratoire d'immunogénétique, F-13005, Marseille, France; b Aix-Marseille Université, CNRS, EFS, ADES UMR 7268, F-13005, Marseille, France; c Centre Hospitalier Universitaire de Besançon, Service d’Ophtalmologie, F-25000, Besançon, France; d Université Marie et Louis Pasteur, UR 4662 Soins Intégrés, Nanomédecine, IA et Ingénierie pour la Santé (SINERGIES), F-25000, Besançon, France; e Nouvel Hôpital Civil CHRU Strasbourg, Service d’Ophtalmologie, F-67000, Strasbourg, France; f Établissement Français du Sang Nouvelle-Aquitaine, Laboratoire d’Histocompatibilité, F-86012, Poitiers, France; g Centre Hospitalier Universitaire de Besançon, Service de virologie, F-25000, Besançon, France; h Établissement Français du Sang Bourgogne Franche-Comté, Activité d'ingénierie cellulaire et tissulaire, F-25000, Besançon, France; i Établissement Français du Sang Nouvelle-Aquitaine, Laboratoire d'ingénierie tissulaire et cellulaire, F-33075, Bordeaux, France; j Inserm U1211 Maladies Rares: Génétique et Métabolisme, Université de Bordeaux, F-33075, Bordeaux, France; k Centre Hospitalier Universitaire de Besançon, Service de Chirurgie Maxillo-Faciale, Stomatologie et Odontologie Hospitalière, F-25000, Besançon, France

**Keywords:** Human amniotic membrane, Biological scaffold, Immunogenicity, HLA-I, HLA-II, HLA-G, Luminex, MAIPA

## Abstract

**Introduction:**

Human amniotic membrane (hAM) is considered to have low immunogenicity since Akle et al. reported no anti-HLA antibodies (Abs) in four volunteers after subcutaneous grafting of fresh hAM in 1981. But the sensitivity of the detection methods has significantly improved since then. Furthermore, hAM graft is a massive local input of allogenic HLA-G whose potential immunogenicity has never been assessed. The objective of this study was to look for the presence of anti-HLA class I, II, and anti HLA-G Abs at 1 and 3 months after hAM transplantation on the ocular surface.

**Material and methods:**

Twenty-three patients who required a hAM graft on the ocular surface (cornea and/or conjunctiva) for any indication were enrolled in this prospective clinical study. Sera were collected on the day of transplantation and at 1- and 3-months post-transplantation (M0, M1, M3). Anti-HLA class I, and II Abs were assessed using the Luminex® Single Antigen technique. Anti HLA-G Abs were assessed using an in-house ELISA-based detection assay, derived from the Monoclonal Antibody specific Immobilization of Platelet Antigen (MAIPA) assay.

**Results:**

Only one patient showed *de novo* Ab apparition after transplantation. Six patients showed a significant increase of preformed Abs MFI (>50 %) during follow up although transient in one out of six. No anti-HLA-G Abs were identified before or after hAM transplantation.

**Conclusion:**

This is the first study to investigate anti-HLA class I, II, and anti HLA-G Abs in a clinical setting after cryopreserved hAM transplantation. The main finding of this study is the minimal humoral immune response in our patients, supporting the immunological safety of the procedure and the excellent clinical tolerance reported across various indications over decades of use.

## Graphical abstract

**Figure:**
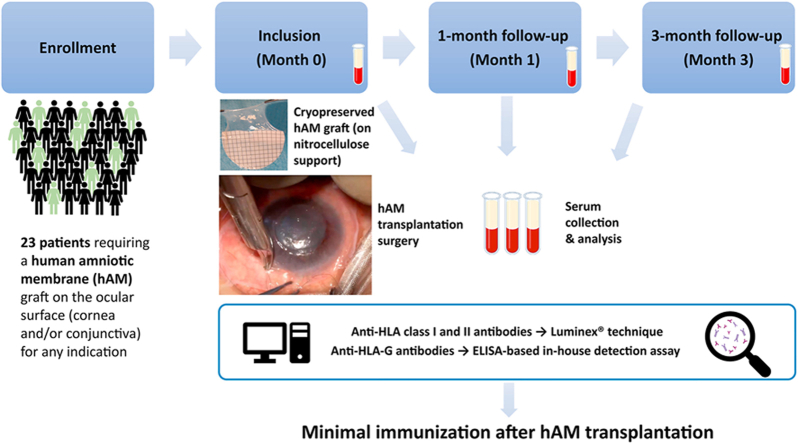

## 1 Introduction

The human amniotic membrane (hAM) is defined as the membrane that forms the wall of an embryo/fetal annex known as the amnion or amniotic sac. This membrane encloses the amniotic cavity and contains amniotic fluid [1]. Histologically, the tissue is composed of a single epithelial layer containing hAM epithelial cells (hAECs), a thick basement membrane, and an avascular stroma containing hAM mesenchymal stromal cells (hAMSCs). It meets the fundamental criteria of tissue engineering scaffolds, being a three-dimensional, biodegradable, and biocompatible matrix enriched with growth factors and stem cells [2,3]. It has been used extensively as a scaffold in clinical tissue engineering applications and has also been developed into advanced formats including hAM-based hydrogels, bioinks, nanoparticles, and hybrid or multilayer composite forms [2,4,5].

The first documented instance of surgical use of hAM was in 1910 by Davis, who employed it as a material for skin grafting [6]. Since then, its applications have expanded significantly to the field of ophthalmology. In 1940, De Roth reported the first use of hAM as a biomaterial for conjunctival reconstruction [7]. Currently, it is commonly used in ophthalmology to reconstruct tissue defects on the ocular surface following the excision of a conjunctival tumor or even corneal perforation, and to treat various ocular pathologies, such as neurotrophic keratitis and pterygium [8,9]. hAM has mechanical, anti-infective, anti-fibrotic, regenerative, and anti-inflammatory properties [10]. When used as a graft, it provides mechanical support during healing and has the ability to reduce scarring and inflammation [10,11]. Its cellular components, cytokines and growth factors also have anti-inflammatory, anti-microbial, and either anti-angiogenic or angiogenic properties (depending on the application), as well as analgesic effects [[10], [11], [12], [13], [14]].

The viability of hAM as a transplantable tissue is well-documented [15,16]. Harvesting and storage procedures are well-established [17]. Furthermore, it has been demonstrated that the usual processes applied to hAM—such as decellularization, de-epithelialization, lyophilization, or dehydration [18]—as well as more advanced processing methods [[19], [20], [21]], significantly impact cell viability, often resulting in the presence of residual DNA, cell-associated proteins, and other cellular debris. It is still unclear whether these processing methods affect the inflammatory reaction.

In routine use, hAM transplantation is well tolerated, with clinical immunological and inflammatory reactions rarely observed. It is said to have low immunogenicity [[22], [23], [24]] with a high margin of clinical safety given the low number of serious adverse reactions relative to the high number of transplants [25,26]. Therefore, little biological testing has been performed to detect hAM immunogenicity, and none is required in clinical practices [14,17,[27], [28], [29], [30]]. The extant literature on the subject is limited to a single study [22]. Akle et al. observed a mild clinical and histological inflammatory reaction following fresh hAM grafts on the skin of seven volunteers. However, the authors were unable to determine whether this reaction was due to healing or to graft rejection. We previously summarized the rare and sometimes contradictory information regarding hAM immunogenicity and the methods used for its detection [31]. Kubo et al. reported the presence of human leukocyte antigens (HLA)-A, -B, -C, and more specifically -G in the hAECs. Additionally, a mild inflammatory reaction was observed in rats following xenotransplantation of hAM [24]. Similarly, Hammer et al. demonstrated that an anti-pan HLA antibody (Ab) strongly reacted with hAECs [32]. In clinical practice, there have been four documented cases of hypopyon after single or multiple hAM grafts from the same donor on the ocular surface in the same patient [[33], [34], [35], [36]], making it possible that an immune reaction occurred although no biological evidence exists.

Since the 1980s and the study of Akle et al., innovative and highly sensitive techniques have been developed to detect anti-HLA Abs. We have extensive experience in HLA detection [[37], [38], [39], [40], [41], [42], [43], [44], [45], [46], [47]] and previously described methods to determine whether antibody-mediated immune response is triggered after hAM grafting in a clinical setting. Techniques such as the Luminex Single Antigen® to detect anti-HLA Class I and II Abs and the modified MAIPA assay (Monoclonal Ab-specific Immobilization of Platelet Antigens, ApDIA®) to detect Abs directed against the non-classical HLA molecule HLA-G provide modern options for assessing hAM immunogenicity [48] since a mild humoral immune response might be induced by hAM allografting, particularly against these antigens (Ags).

The aim of our study was to assess the immunogenicity of hAM transplantation on the ocular surface with modern sensitive techniques for detecting anti-HLA Abs.

## 2 Materials and methods

### Ethics approval

Ethics approval for the study was obtained from the “Comité de protection des personnes Sud-Est I″ committee (2023-A01447-38). Patients were enrolled in the study after providing free and informed written and oral consent.

### 2.1 Study design

This was a prospective cohort study conducted at the University Hospital of Besançon in the Department of Ophthalmology between August 2022 and September 2023, in collaboration with the French Blood Establishment of Bourgogne Franche-Comté (BFC), the French Blood Establishment of Nouvelle-Aquitaine (EFS NAq) and the French Blood Establishment of Provence Alpes Côte d'Azur (EFS PACA).

### 2.2 Eligibility criteria

The inclusion criteria for the study were as follows: patients over the age of 18 who could consent to the study protocol and who required a hAM graft on the ocular surface (cornea and/or conjunctiva) for any indication.

Patients were excluded if they met any of the following criteria: under the age of 18, pregnant or had given birth less than 6 months prior, history of organ transplantation, had received blood products within the last 6 months, were receiving immunosuppressive or immunomodulatory treatments, or were unable to provide consent or refused to participate in the study protocol.

### 2.3 Cohort characteristics

A comprehensive analysis of the patients' medical records was conducted to collect relevant data, including age, gender, medical history, parity, gestational age for women, vaccinations, transfusions, treatments, surgical indication, surgery date, surgery type, and clinical changes over the 3-month postoperative follow-up period. The occurrence of local inflammatory reactions or graft failure was meticulously documented.

### 2.4 hAM preservation and preparation

The hAM was cryopreserved and obtained from AICT bank from EFS BFC at Besançon. A piece of hAM 4.7 cm in diameter was transported with dry ice blocks to ensure it stayed frozen at −80 °C. The allografts were stored in glycerol on a nitrocellulose support, with the epithelial side facing the support. Upon receipt, they were thawed for 2 h at room temperature followed by three, 5 min rinses in saline injection solution.

### 2.5 Surgical procedures and follow-up

Surgery was performed under local or general anesthesia. Different surgical techniques (multilayer, inlay, overlay) were used depending on the surgical indication. All patients were treated with anti-inflammatory eye drops and ointments for 1 month after the surgery. Postoperative visits were scheduled at 15 days, 1 month, and 3 months to monitor signs of rejection or unusual local inflammatory reactions and to ensure successful treatment of the initial pathology.

Study visits were planned at baseline (M0), one month (M1), and three months (M3), with interim checks conducted at the clinician's discretion.

### 2.6 Blood collection

Three blood samples were collected. The first (M0) was taken in the operating room prior to hAM transplantation and served as a reference to identify any pre-existing anti-HLA Abs in the graft recipients, which could have developed during pregnancy or prior immunization. A second blood sample was taken at 1 month (M1), corresponding to the hypothetical reappearance of preformed anti-HLA Abs after stimulation. A third sample was scheduled at 3 months (M3) to study the kinetics of anti-HLA Abs, to confirm their presence and assess *de novo* immunization. This 3-months follow-up period is consistent with immunological surveillance recommendations after exposure to biological materials. For example, French regulations mandate the screening for irregular red cell Abs (agglutinins) 3 months after the last blood transfusion. Similarly, French national expert society [49] recommends reassessing the anti-HLA Ab profile 3 weeks after a blood transfusion (in context of pre Hematopoietic Stem Cell Transplantation). hAM grafts are typically resorbed within a maximum of 4 weeks [50]. Given the rapid and transient nature of the graft, it is unlikely that the tissue would elicit a delayed immune response if one has not occurred during the first three months. Blood samples were collected in a dry tube (5–7 mL), centrifuged, and decanted. Sera were then stored at −20 °C in a serum bank until analysis.

### 2.7 Anti-HLA class I, II and G analysis

Anti-HLA class I and II Abs detection was performed using Luminex® Single Antigen equipment at the EFS NAq and the EFS PACC laboratories. Briefly, the samples were incubated with a mixture of fluorescent polystyrene microbeads, each bead being coated with a high density of a single HLA Ag. The beads were washed through a filter plate. Then, Ab binding was revealed by fluorescent anti IgG secondary Ab. The bead suspension was analyzed by a Luminex® instrument. Each bead was identified through its intrinsic fluorescence. The signal linked to the quantity of Abs fixed on it was quantified as Mean Fluorescence Intensity (MFI) [51]. A MFI superior to 1000 was considered a positive result. This technique is particularly sensitive and can detect a specific signal as low as 500 of MFI. Around 100 HLA class I Ags and 100 HLA class II Ags were tested by the kit with very high sensitivity. These Ags correspond to the most frequent HLA alleles in the European population. This technique is the international reference method for anti-HLA Ab detection in medical contexts such as HSCT or solid organ transplantation [52]. For each patient and timepoint, we calculated Virtual Panel Reactive Abs (vPRA) using Eurotransplant's Virtual PRA Calculator Online Tool at the 1000 MFI threshold. The vPRA is based on the European HLA typing database. For each patient, vPRA was determined for class I and class II Abs at M0, M1 and M3. In addition, a qualitative analysis of the Ab profiles was performed. Abs profiles were independently reviewed by two expert biologists from our laboratory to determine *de novo* Abs apparition. Then, Abs MFI data were reviewed to determine if significant variations of the existing Abs MFI occurred during the post-transplantation period. For preformed Abs, a 50 % variation of MFI between M0 and M1 or M3 was considered significant as previously established [53].

In order to detect anti-HLA-G Abs, we modified the MAIPA kit (ApDIA®) so to detect Abs directed against HLA-G associated with β2m. [54]. This kit is based on the ELISA principle and allows the detection and identification of auto/allo anti-platelet glycoprotein Abs and anti-classical class I HLA Abs in serum using a platelet panel and platelet-Ag immobilized by a monoclonal Ab. Its sensitivity is similar to Luminex® Single Ag for the detection of classical HLA class I Abs [54]. All the MAIPA reagents were unchanged for our modified protocol except the platelet panel which was replaced by HLA-G transfected K562 cells and the capture Ab which was replaced by MEM-G9 (MA1-19014, Invitrogen). Briefly, patient serum was incubated with HLA-G–transfected cells, allowing the formation of a cell–patient anti-HLA-G Ab complex. After incubation with MEM-G/9 (mouse anti HLA-G), a tripartite complex (MEM-G/9–cell–patient anti-HLA-G Ab) forms. Following cell lysis, this tripartite complex was captured on the ELISA plate by a goat anti-mouse Ab recognizing MEM-G/9. A goat anti-human Ig HRP Ab was used to specifically detect patient Abs.

This method has been validated for anti-HLA-G Ab detection and published in a lung transplant cohort [48]. Each serum was tested in parallel on wild-type K562 cells and HLA-G transfected K562 cells. Each serum was first tested by a screening MAIPA using a pool of three transfected cell lines (HLA-G∗01:01, HLA-G∗01:04 and HLA-G∗01:06 cells). Serum samples were flagged as suspicious based on the optical density (OD) value obtained, taking into account the OD obtained with the corresponding wild-type control well. Suspicious samples (combination of OD > 0.1 and OD (HLA-G)/OD (wild type) ratio >2) were checked by an identification MAIPA using G101, G104 and G106 cells separately to increase sensitivity to a particular HLA-G molecule and to determine Ab specificity. The use of non-transfected K562 cells allowed us to evaluate the background noise of our system. Each plate included the positive and negative control sera of the MAIPA kit and a in-house positive control, tested on transfected and untransfected cells, as well as two blank wells. The assay was considered valid if the OD of the negative controls was <0.1 and if the OD of the positive controls was >0.2. Given that our test mimics the steps of the MAIPA assay, each plate included the MAIPA kit platelet control pool and served as an internal control for the technical steps (incubation, washing, flicking). The OD of the platelet control pool had to be within the kit's expected values to validate the entire analytical process.

### 2.8 Outcome measures

The primary outcome measure was the presence and identification of Class I, II and G anti-HLA Abs in serum samples at M1 and M3 after hAM transplantation on the ocular surface.

### 2.9 Statistical analysis

Descriptive statistics were applied to analyze numerical and nominal data, while inferential statistics were used to assess differences in MFI and PRA from M0 to the M1 and M3 follow-up visits.

## 3 Results

### 3.1 Patient characteristics

Twenty-three patients who underwent hAM transplantation were enrolled between August 2022 and September 2023. A total of 69 serum samples were collected at M0, M1 and M3.

**Fig. 1:**
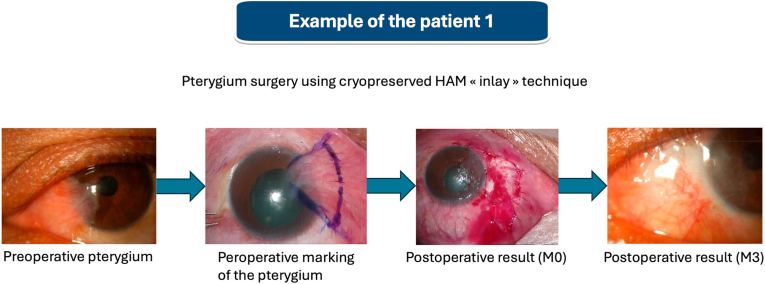
An example of pre, intra- and postoperative clinical findings after pterygium surgery using cryopreserved human amniotic membrane (hAM) “inlay” technique in patient 1.

The characteristics of the cohort are presented in Table 1. The patient population was predominantly male (65 %). The median age was 62 years old (55 years old for male participants and 66.5 years old for female participants). The most frequent indication for hAM was pterygium surgery with an inlay graft technique (20 patients, 87 %). Four patients (17 %) had a history of transfusion. Six patients (26 %) had a history of pregnancy. Nine patients (39 %) had undergone an immunizing event prior to hAM transplantation (Fig. 1).

**Table 1:** Description of the characteristics of the study cohort (M Male, F Female, NA not applicable).

Patient	Age	Sex	Indication	Graft surgical technique	Location	Transfusion	Pregnancy
1	72	M	Pterygium	Inlay	Conjunctiva	Yes (In 1992)	NA
2	77	M	Pterygium	Inlay	Conjunctiva	Yes (In 2013)	NA
3	81	F	Corneal ulcer	Sandwich	Conjunctiva-cornea	No	5 times
4	76	M	Pterygium	Inlay	Conjunctiva	No	NA
5	56	M	Pterygium	Inlay	Conjunctiva	No	NA
6	50	M	Pterygium	Inlay	Conjunctiva	No	NA
7	74	M	Pterygium	Inlay	Conjunctiva	No	NA
8	26	M	Pterygium	Inlay	Conjunctiva	No	NA
9	48	F	Rosacea	Inlay	Conjunctiva	No	0
10	52	M	Pterygium	Inlay	Conjunctiva	Yes (In 1990)	NA
11	55	M	Pterygium	Inlay	Conjunctiva	No	NA
12	74	M	Corneal ulcer	Sandwich	Conjunctiva-cornea	No	NA
13	62	M	Pterygium	Inlay	Conjunctiva	No	NA
14	73	F	Pterygium	Inlay	Conjunctiva	No	3 times
15	77	F	Pterygium	Inlay	Conjunctiva	Yes (In 1971)	5 times
16	32	F	Conjunctival naevus	Inlay	Conjunctiva	No	0
17	71	F	Pterygium	Inlay	Conjunctiva	No	6 times
18	44	M	Pterygium	Inlay	Conjunctiva	No	NA
19	62	F	Pterygium	Inlay	Conjunctiva	No	2 times
20	62	F	Pterygium	Inlay	Conjunctiva	No	3 times
21	49	M	Pterygium	Inlay	Conjunctiva	No	NA
22	55	M	Pterygium	Inlay	Conjunctiva	No	NA
23	33	M	Pterygium	Inlay	Conjunctiva	No	NA

During the 3-month follow-up, no major local inflammatory reaction or clinical rejection were observed.

### 3.2 Anti-HLA class I, II and G analysis

**Fig. 2:**
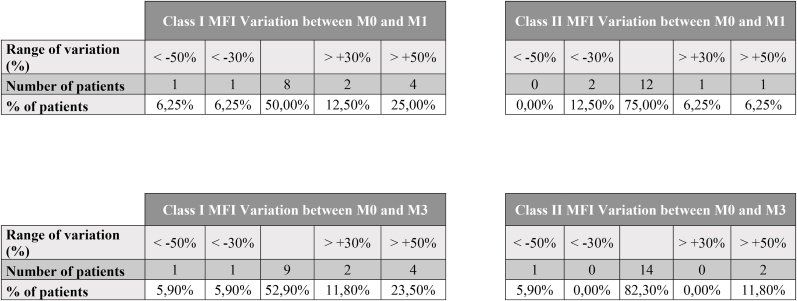
Distribution of patients according to the range of MFI variation of the immunodominant Ab. The figure shows the number of patients in each MFI variation range and the corresponding percentage of patients within the sensitized cohort between M0 and M1 (n = 16) and between M0 and M3 (n = 17).

Abs assigned at the MFI threshold of 1000, along with their respective MFI values, are summarized in Table 2. With the exception of the appearance of B82 in patient 17, no *de novo* Ab development was observed in our patients. Five patients showed a significant increase (>50 %) of MFI for preformed Abs between M0 and M3: patients 4, 13 and 16 for class I Abs; patient 20 for class II Abs; patient 23 for both class I and II Abs. For patient 4, 13 and 23, the MFI increases were already significant by M1 contrary to patient 16 and 20. In addition, Patient 14 exhibited a transient significant increase of class I Abs MFI at M1 that regressed by M3 (Fig. 2).

**Table 2:** Patients immunization follow-up based on Luminex Single Antigen (One lambda) results. Antibody specificities and MFI (Mean Fluorescence Intensity) are displayed for each timepoint. Positivity threshold is 1000 of MFI. Antibodies with MFI >1000 are highlighted in bold and were used for vPRA calculation. Antibodies which did not exceed an MFI of 1000 at all time points, are reported but not highlighted in bold.

Patient	HLA class	Anti-HLA Abs specificity (and MFI) at M0	Anti-HLA Abs specificity (and MFI) at M1	Anti-HLA Abs specificity (and MFI) at M3
**Patient 1**	Class I	Neg	Neg	Neg
	Class II	Neg	Neg	Neg
**Patient 2**	Class I	Neg	Neg	Neg
	Class II	Neg	Neg	Neg
**Patient 3**	Class I	Neg	Neg	Neg
	Class II	Neg	Neg	Neg
**Patient 4**	Class I	**B45 (1710)** B50 (440)	**B45 (2690)** B50 (810)	**B45 (4520)** **B50 (2310)**
	Class II	Neg	Neg	Neg
**Patient 5**	Class I	**B46 (1570)**	**B46 (1510)**	**B46 (1550)**
	Class II	Neg	Neg	Neg
**Patient 6**	Class I	**A∗11:02 (2260)**	**A∗11:02 (3390)**	**A∗11:02 (2880)**
	Class II	**DRB5∗01:01 (1700)**	**DRB5∗01:01 (1150)**	**DRB5∗01:01 (1720)**
**Patient 7**	Class I	**B45 (2350)**	**B45 (2750)**	**B45 (1740)**
	Class II	Neg	Neg	Neg
**Patient 8**	Class I	**A26 (1880)** **A43 (1610)** **A66 (1510)** **A25 (1300)** **Cw17 (1920)**	A26 (920) A43 (690) A66 (560) A25 (790) Cw17 (980)	**A26 (1550)** **A43 (1270)** **A66 (1160)** **A25 (1180)** **Cw17 (1790)**
	Class II	Neg	Neg	Neg
**Patient 9**	Class I	A66 (660) B8 (680) **Cw17 (3510)**	A66 (850) **B8 (1040)** **Cw17 (4660)**	**A66 (1030)** B8 (980) **Cw17 (4300)**
	Class II	Neg	Neg	Neg
**Patient 10**	Class I	A25 (990) **B75 (2950)**	**A25 (1130)** **B75 (2540)**	A25 (620) **B75 (1910)**
	Class II	**DQA1∗03 (2170)** **DP19 (3160)**	**DQA1∗03 (2190)** **DP19 (3460)**	**DQA1∗03 (2020)** **DP19 (3170)**
**Patient 11**	Class I	**Cw1 (1150)**	Cw1 (960)	Cw1 (210)
	Class II	Neg	Neg	Neg
**Patient 12**	Class I	B82 (820)	B82 (930)	**B82 (1160)**
	Class II	Neg	Neg	Neg
**Patient 13**	Class I	B63 (640)	B63 (970)	**B63 (1020)**
	Class II	Neg	Neg	Neg
Patient 14	Class I	A24 (4490) A23 (3780) A2 (610) A69 (400) A68 (350) B48 (5620) B60 (2370) B81 (820)	A24 (8670) A23 (7610) A2 (1520) A69 (1220) A68 (1140) B48 (8600) B60 (4080) B81 (1590)	A24 (6260) A23 (5350) A2 (1040) A69 (750) A68 (700) B48 (6560) B60 (3300) B81 (1240)
	Class II	DR9 (4320) DR4 (3360) DR18 (2420) DR52 (2460) DR17 (1940) DR13 (1200) DR11 (1150) DR14 (900) DR8 (770) DR7 (1180) DQ7 (6790) DQ9 (4900) DQ8 (5080) DQ4 (880)	DR9 (4400) DR4 (3870) DR18 (3560) DR52 (2930) DR17 (2820) DR13 (1510) DR11 (1430) DR14 (1300) DR8 (1170) DR7 (1090) DQ7 (8600) DQ9 (6190) DQ8 (6060) DQ4 (1310)	DR9 (2820) DR4 (2140) DR18 (1740) DR52 (1700) DR17 (1510) DR13 (840) DR11 (770) DR14 (600) DR8 (540) DR7 (630) DQ7 (5830) DQ9 (4380) DQ8 (4480) DQ4 (620)
**Patient 15**	Class I	NA	**B57 (1520)**	**B57 (1780)**
	Class II	NA	DR9 (940)	**DR9 (1180)**
**Patient 16**	Class I	**A1 (5120)** **A30 (4410)** **A3 (2180)** **A31 (1990)** **A36 (1520)** **A32 (1350)** **A11 (1290)** **A74 (1210)** **B73 (5540)** B47 (230) Cw18 (930)	**A1 (6620)** **A30 (6010)** **A3 (2790)** **A31 (2280)** **A36 (2000)** **A32 (1310)** **A11 (1240)** **A74 (1410)** **B73 (7060)** B47 (470) **Cw18 (1540)**	**A1 (8030)** **A30 (7330)** **A3 (4270)** **A31 (4010)** **A36 (2970)** **A32 (2530)** **A11 (3120)** **A74 (2490)** **B73 (7570)** **B47 (1160)** **Cw18 (1690)**
	Class II	Neg	Neg	Neg
**Patient 17**	Class I	**A80 (3420)** **B37 (1420)** B82 (200) **Cw17 (1560)**	**A80 (2620)** **B37 (1170)** B82 (480) **Cw17 (1170)**	**A80 (2830)** B37 (900) **B82 (2330)** Cw17 (980)
	Class II	**DR7 (2340)** **DR53 (2430)**	**DR7 (1660)** **DR53 (2190)**	DR7 (900) **DR53 (1920)**
**Patient 18**	Class I	B76 (570)	**B76 (1270)**	B76 (630)
	Class II	Neg	Neg	Neg
Patient 19	Class I	A33 (3800) A66 (2220) Bw6 (14200) B51 (4970) B52 (1370) B59 (1230) Cw5 (10320) Cw18 (1580) Cw6 (1020)	NA	A33 (3470) A66 (1920) Bw6 (13770) B51 (4430) B52 (1230) B59 (1160) Cw5 (9510) Cw18 (1360) Cw6 (930)
	Class II	DR11 (13450) DRB1∗13:03 (12240) DR8 (10520) DR4 (5610) DQ7 (>20000) DQ9 (18610) DQ8 (18120) DQ2 (14920) DQ4 (10870)	NA	DR11 (13040) DRB1∗13:03 (11440) DR8 (10460) DR4 (5240) DQ7 (>20000) DQ9 (17770) DQ8 (17820) DQ2 (14850) DQ4 (11330)
**Patient 20**	Class I	**B53 (17760)** **B35 (17710)** **B51 (15660)** **B78 (14490)** **B75 (9090)** **B18 (3740)** **Cw17 (1040)**	**B53 (17910)** **B35 (18880)** **B51 (16230)** **B78 (14710)** **B75 (8140)** **B18 (2770)** Cw17 (740)	**B53 (15920)** **B35 (17250)** **B51 (14300)** **B78 (13220)** **B75 (7640)** **B18 (2980)** Cw17 (670)
	Class II	DQ8 (730) DQ9 (610)	DQ8 (610) DQ9 (440)	**DQ8 (1210)** **DQ9 (1690)**
**Patient 21**	Class I	Neg	Neg	Neg
	Class II	Neg	Neg	Neg
**Patient 22**	Class I	**B75 (4650)**	**B75 (3970)**	**B75 (4310)**
	Class II	**DR1 (1120)** **DR52 (2750)**	**DR1 (1610)** **DR52 (3870)**	**DR1 (1570)** **DR52 (3330)**
**Patient 23**	Class I	**Cw17 (1280)**	**Cw17 (2800)**	**Cw17 (2000)**
	Class II	DR53 (650) **DQ9 (1460)** **DQ8 (1450)**	**DR53 (1140)** **DQ9 (6880)** **DQ8 (6900)**	DR53 (600) **DQ9 (2650)** **DQ8 (2690)**

vPRA data are summarized in Table 3. Significant disparity was observed in the initial PRAs, with higher values recorded for the female participants compared to the male ones (mean 32.7 % vs 5.9 %, respectively; p = 0.043). For some patients, important variations of vPRA values occurred during follow up. The most noticeable variations involved antibodies near the positivity threshold (MFI: 1000), which fluctuated around this cutoff from one sample to another.

**Table 3:** Patients immunization follow-up based on class I and class II virtual Panel Reactive Antibody (vPRA, Eurotransplant, threshold used for calculation: MFI >1000) before transplantation (M0), at 1 month postoperative (M1) and 3 months postoperative (M3) (∗no significant modification of the specificities, NA non-available data, M Male, F Female).

Patient	Gender	PRA	M0	M1	M3	Patient	Gender	PRA	M0	M1	M3
Patient 1	M	PRA class I	0,00 %	0,00 %	0,00 %	Patient 13	M	PRA class I	0,00 %	0,00 %	0,87 %
		PRA class II	0,00 %	0,00 %	0,00 %			PRA class II	0,00 %	0,00 %	0,00 %
Patient 2	M	PRA class I	0,00 %	0,00 %	0,00 %	Patient 14	F	PRA class I	29,00 %	71,93 % ∗	67,26 %
		PRA class II	0,00 %	0,00 %	0,00 %			PRA class II	90,20 %	92,08 %	84,06 %
Patient 3	F	PRA class I	0,00 %	0,00 %	0,00 %	Patient 15	F	PRA class I	NA	6,62 %	6,62 %
		PRA class II	0,00 %	0,00 %	0,00 %			PRA class II	NA	0,00 %	1,83 %
Patient 4	M	PRA class I	0,84 %	0,84 %	3,2 % ∗	Patient 16	F	PRA class I	69,10 %	69,12 %	69,16 %
		PRA class II	0,00 %	0,00 %	0,00 %			PRA class II	0,00 %	0,00 %	0,00 %
Patient 5	M	PRA class I	0,04 %	0,04 %	0,04 %	Patient 17	F	PRA class I	4,76 %	4,76 %	1,88 % ∗
		PRA class II	0,00 %	0,00 %	0,00 %			PRA class II	44,80 %	44,80 %	40,05 %
Patient 6	M	PRA class I	0,00 %	0,00 %	0,00 %	Patient 18	M	PRA class I	0,00 %	0,00 %	0,00 %
		PRA class II	23,84 %	23,84 %	23,84 %			PRA class II	0,00 %	0,00 %	0,00 %
Patient 7	M	PRA class I	0,84 %	0,84 %	0,84 %	Patient 19	F	PRA class I	97,12 %	NA	93,92 %
		PRA class II	0,00 %	0,00 %	0,00 %			PRA class II	81,78 %	NA	81,78 %
Patient 8	M	PRA class I	12,50 %	0 % ∗	12,50 %	Patient 20	F	PRA class I	40,60 %	39,16 %	39,16 %
		PRA class II	0,00 %	0,00 %	0,00 %			PRA class II	0,00 %	0,00 %	25 % ∗
Patient 9	F	PRA class I	1,83 %	21,96 % ∗	2,11 %	Patient 21	M	PRA class I	0,00 %	0,00 %	0,00 %
		PRA class II	0,00 %	0,00 %	0,00 %			PRA class II	0,00 %	0,00 %	0,00 %
Patient 10	M	PRA class I	0,08 %	4,08 % ∗	0,08 %	Patient 22	M	PRA class I	0,08 %	0,08 %	0,08 %
		PRA class II	27,58 %	27,58 %	27,58 %			PRA class II	77,40 %	77,40 %	77,40 %
Patient 11	M	PRA class I	7,41 %	0 % ∗	0,00 %	Patient 23	M	PRA class I	1,83 %	1,83 %	1,83 %
		PRA class II	0,00 %	0,00 %	0,00 %			PRA class II	25,00 %	45 % ∗	25,00 %
Patient 12	M	PRA class I	0,00 %	0,00 %	0,00 %						
		PRA class II	0,00 %	0,00 %	0,00 %						

### 3.3 Anti-HLA-G analysis

OD results for our adapted MAIPA-assay are summarized in Table 4. No anti HLA-G Abs were detected in the cohort's serum samples, neither prior to transplantation (M0) nor at M1 or M3. Patient 7 at M0 and patients 10 and 11 at M1 had suspicious results but these results were not confirmed by the identification MAIPA assay.

**Table 4:** Optical density (OD) values for modified MAIPA assay before transplantation (M0), at 1 month postoperative (M1) and 3 months postoperative (M3) on Wild Type cells and HLA-G transfected pooled cells (∗not confirmed, NA non-available data).

Patient	M0	M1	M3	Patient	M0	M1	M3
1	0,035	0,026	0,045	13	0,082	0,021	0,016
	0,029	0,018	0,014		0,085	0,044	0,026
2	0,089	0,017	0,020	14	0,124	0,031	0,027
	0,035	0,015	0,020		0,107	0,038	0,037
3	0,079	0,068	0,040	15	NA	0,031	0,012
	0,129	0,027	0,030		NA	0,069	0,022
4	0,099	0,012	0,063	16	0,083	0,033	0,058
	0,111	0,017	0,066		0,089	0,036	0,042
5	0,049	0,104	0,021	17	0,138	0,045	0,028
	0,065	0,020	0,019		0,129	0,029	0,020
6	0,159	0,010	0,091	18	0,053	0,054	0,028
	0,114	0,016	0,105		0,092	0,060	0,026
7	0,136	0,022	0,031	19	0,080	NA	0,135
	**0,264∗**	0,024	0,063		0,056	NA	0,098
8	0,031	0,019	0,024	20	0,015	0,060	0,093
	0,033	0,034	0,062		0,036	0,063	0,036
9	0,089	0,063	0,038	21	0,065	0,070	0,158
	0,169	0,041	0,052		0,078	0,052	0,093
10	0,387	0,059	0,097	22	0,111	0,060	0,030
	0,120	**0,125∗**	0,074		0,071	0,095	0,035
11	0,054	0,105	0,081	23	0,075	0,014	0,032
	0,102	**0,314∗**	0,021		0,085	0,011	0,044
12	0,049	0,021	0,068				
	0,067	0,055	0,096				

OD on Wild Type cells.

OD on HLA-G transfected cells pool.

## 4 Discussion

This is the first study to describe anti-HLA-I, -II and -G antibodies in a clinical setting of hAM transplantation using highly sensitive methods. The hAM used for grafting in our 23 patients were not specifically tested for their expression of classical HLA or HLA-G before the transplantation. However, the expression of these Ags by amniotic membranes, including after processing and preservation, has been well demonstrated in previous studies [24,32].

### 4.1 Classic anti-HLA class I and II Abs

In most patients (16/23), no change in the profile of anti-classical HLA Abs was observed at M1 or M3 post-transplantation compared to M0, despite using a high-sensitivity detection technique (Table 2). *De novo* Abs apparition occurred in only one patient: patient 17 developed anti HLA-B82 Abs at M3. But B82 in an extremely rare Ag in the general population making it unlikely to represent a specific reaction against the donor.

The present study has an important limitation regarding the interpretability of the results: we were unable to retrieve the HLA typing of the placentas used for hAM recovery. Therefore, we could not determine donor-specific Abs (DSA). Instead, we relied on careful analysis of the MFI data at each timepoint and on the vPRA. Although the vPRA is routinely used in transplantation as a reflection of the patient's overall immunization status, it has some limitations. For instance, it may remain unchanged despite an increase in MFI values or, on the contrary, change dramatically according to the MFI cut-off used for calculation. The apparent vPRA variation seen in 9 patients (Table 3: patients 4, 8, 9, 10, 11, 14, 17, 20, and 23) can be explained by low MFI Abs that were fluctuating slightly below or above the 1000 MFI threshold depending on the timepoint. Therefore, no significant variation in vPRA was observed over time in any of the 23 patients.

Interestingly, Patients 4, 13, 16, 20 and 23 had a significant increase of performed Abs MFI value between M0 and M3. Also, patient 14 showed a transient increase of the MFI which was not confirmed at M3.(Fig. 2). These results are difficult to interprete in the absence of the hAM typings. It could be either an increase following a specific stimulation of preformed Abs by the graft or, since these Abs are preformed, the increase could reflect non-specific external factors such as the patient's hydration status at the time of sampling or an inflammatory state which can boost all preformed Abs non-specifically. In these patients, we observe a global increase of the MFI of all the preformed Abs, which is not in favor of a specific stimulation, especially when the MFI increase in transient. The Luminex® Single Antigen method is known for having an important coefficient of variation, especially for low MFI results which is the case for Patient 13 and 20. In addition, patients with high levels of immunization such as Patient 14 or 16 frequently display MFI fluctuations during long term follow-up for example on organ transplant waiting list. Class II Abs MFI elevation observed in Patients 20 and 23 also seems unrelated to the hAM itself, since this tissue lacks the expression of HLA class II antigens [55].

Therefore, given that no local inflammation or adverse event was reported during the 3 months follow-up period, the Abs profile changes noted in some patients don't apparently have a clinical relevance in the context of hAM transplantation on the ocular surface. However, the observed increases in MFI may have implications for a subsequent organ transplantation. For example, in France, Abs with an MFI exceeding 2000 are considered a contraindication for transplantation with a graft expressing the corresponding antigen.

Thus our results, showing minimal anti HLA humoral response corroborate and update the findings from the 1980s [22]. The low expression of classical HLA molecules by the hAM and the low vascularization of the graft are widely accepted explanations for the lack of immunization in this context, despite the otherwise high immunogenicity of HLA molecules [9,24]. The abundant presence of HLA-G in the hAM graft likely induces local immune tolerance. The immunomodulatory properties of HLA-G are well documented. Binding of HLA-G to human inhibitory receptors Ig-like transcript 2 and 4 (ILT2 and ILT4) receptors, which are expressed by a wide range of immune effectors, induces phenotypic changes and lymphocyte anergy [56]. A tolerogenic microenvironment forms around cells expressing HLA-G, which benefits patients in the context of corneal and organ transplantation but harms them in tumor settings [57,58].

### 4.2 Anti-HLA-G Abs

Since hAM transplantation is a rare instance of encountering allogeneic HLA-G outside of pregnancy, it is a fascinating opportunity to explore the possibility of alloimmunization against graft-derived HLA-G. This assertion is substantiated by a recent report, which provides the first description of anti-HLA-G Abs [59]. The transduced cells used in our study do not express classical HLA Ags, as confirmed by flow cytometry. Consequently, the possibility of interference due to the presence of anti-classical HLA Abs, which may be present in sera, can be ruled out.

Yet, no anti HLA-G Ab was confirmed in our cohort, neither prior to transplantation (M0) nor at M1 or M3. The signal detected in two patients at M1 post-transplantation could have been indicative of an alloimmune response, emerging 1 month after the immunizing event. Conversely, the observed attenuation of the signal at M3 was in favor of weak alloimmunization, which falls below the limits of detection over time. However, these results were not confirmed by the identification test, thereby supporting the validity of our strategy, which entailed a screening step with pooled cells followed by a specific identification step to confirm the findings.

HLA-G has a significantly lower degree of polymorphism compared to classic class I HLA. The selected cell lines were chosen specifically to allow detection of the vast majority of anti-HLA-G Abs. Indeed, HLA-G∗01:01, ∗01:04, and ∗01:06 are the predominant haplotypes, accounting for 81 % of all known haplotypes and 88.5 % of those found in the European population [60]. Notably, HLA-G∗01:01 alone represents 80 % of European haplotypes. It is therefore unlikely that our negative results are due to the detection system being limited to only three HLA-G alleles. However, alloimmunization can only occur in the presence of a HLA-G mismatch between the donor and the patient. A mismatch is statistically improbable in our cohort, which further reduces the likelihood of a false-negative result [61]. A limitation of our study is therefore the absence of HLA-G genotyping data for placenta donors and patients. This information would have strengthened the alloimmunization hypothesis [60].

Recently, the detection of Abs against HLA-G in autoimmune diseases has led to the hypothesis that anti-HLA-G auto-Abs may exist [59]. This possibility could explain the non-confirmed reactions observed in a small number of our patients. Indeed, autoAbs—such as anti-platelet autoAbs detected using MAIPA, from which our detection technique is derived—are characterized by weak and fluctuating signals between samples. Additionally, these materials have heightened sensitivity to freeze–thaw cycles. The sera in our cohort underwent multiple freeze–thaw cycles prior to HLA-G analysis. In particular, sera that tested positive in the screening phase were frozen again before the identification test, which could explain the loss of signal.

Subsequent studies with well-controlled pre-analytical conditions will be necessary to confirm or refute the existence of anti-HLA-G Abs in this and other (transplantation, pregnancy, etc.) clinical settings. In a previous study, our attempts to detect anti-HLA-G Abs in a cohort of lung transplant recipients were unsuccessful. These Abs, whether classified as auto- or alloAbs, could have pathological implications by impeding HLA-G interaction with its receptors, thereby reducing its inhibitory functions.

## 5 Conclusion

Given the advances in the knowledge about HLA expression by the hAM and in detection methods sensitivity, it was important to confirm previous results in a larger cohort. This is the first study to investigate anti-HLA class I, II, and anti HLA-G Abs in a clinical setting after cryopreserved hAM transplantation using high-sensitivity methods. Our results corroborate and modernize those obtained in the 1980s. The main finding of this study is the minimal humoral immune response regarding HLA class I and II and the absence of anti-HLA-G Abs development in patients who underwent this procedure. Our findings align with our experience of excellent clinical tolerance of the hAM used on the ocular surface.

## CRediT authorship contribution statement

**Jean-Baptiste Baudey:** Writing – review & editing, Writing – original draft, Visualization, Validation, Resources, Methodology, Investigation, Formal analysis, Data curation, Conceptualization. **Lauriana Solecki:** Writing – review & editing, Writing – original draft, Visualization, Validation, Supervision, Resources, Methodology, Investigation, Funding acquisition, Formal analysis, Data curation, Conceptualization. **Christophe Picard:** Visualization, Validation, Resources, Methodology, Investigation, Funding acquisition, Conceptualization. **Bastien Mathéaud:** Resources, Methodology, Investigation, Formal analysis, Data curation, Conceptualization. **Pauline Jamain:** Validation, Resources, Methodology, Investigation, Formal analysis, Data curation, Conceptualization. **Isabelle Jollet:** Methodology, Investigation, Formal analysis, Data curation, Conceptualization. **Lucas Hubert:** Methodology, Investigation, Formal analysis, Data curation, Conceptualization. **Alain Coaquette:** Methodology, Investigation, Formal analysis, Conceptualization. **Adeline Desanlis:** Methodology, Formal analysis, Conceptualization. **Xavier Lafarge:** Visualization, Supervision, Methodology, Investigation, Funding acquisition, Formal analysis, Data curation, Conceptualization. **Pascal Pedini:** Visualization, Validation, Supervision, Resources, Methodology, Investigation, Funding acquisition, Formal analysis, Data curation, Conceptualization. **Florelle Gindraux:** Writing – review & editing, Writing – original draft, Visualization, Validation, Supervision, Resources, Project administration, Methodology, Investigation, Funding acquisition, Formal analysis, Data curation, Conceptualization.

## Funding

Call for proposals 2023, French Agency of Biomedicine.

## Declaration of competing interest

The authors declare that they have no known competing financial interests or personal relationships that could have appeared to influence the work reported in this paper.

## Data Availability

Data will be made available on request.
